# Mortality among male cigar and cigarette smokers in the USA

**DOI:** 10.1186/s12954-020-00446-4

**Published:** 2021-01-07

**Authors:** Brad Rodu, Nantaporn Plurphanswat

**Affiliations:** 1 James Graham Brown Cancer Center, University of Louisville, 505 South Hancock Street, Louisville, KY 40202 USA; 2 Department of Medicine, School of Medicine, University of Louisville, Louisville, USA

**Keywords:** Cigar smoking, Cigarette smoking, National health interview survey, Mortality

## Abstract

**Background:**

Cigars and cigarettes are both smoked, but much less is known about the former’s long-term health effects, due to its low prevalence and infrequent collection of cigar information in national surveys.

**Purpose:**

We conducted a follow-up mortality study of cigar-smoking men age 40–79 years in National Health Interview Surveys (NHIS).

**Methods:**

We used pooled NHIS files linked to the National Death Index to obtain follow-up from year of interview to year of death or December 31, 2015. We developed categories of cigarette and cigar smoking that accommodate dual and former use of both products. We used Cox proportional hazards models, adjusted for age, race/ethnicity, marital status, education, income and region to estimate hazard ratios (HRs, 95% confidence intervals, CI) for mortality from all causes, heart diseases, malignant neoplasms, cerebrovascular disease, chronic lower respiratory diseases and two mutually exclusive categories: smoking-related and other diseases.

**Results:**

There were 14,657 deaths from all causes, including 3426 never tobacco users, 3276 exclusive cigarette smokers and 176 exclusive cigar users. The latter had no statistically significant evidence of increased mortality from all causes, heart diseases, malignant neoplasms, cerebrovascular disease, smoking-related diseases or other causes. In contrast, the mortality experience of dual users of cigars and cigarettes and cigar smokers who formerly used cigarettes is similar to exclusive cigarette smokers.

**Conclusions:**

This study provides evidence that male cigar smokers age 40 + years had elevated mortality risks. However, after accounting for cigarette smoking and other confounding variables, we found significantly increased mortality only among dual and former users of cigarettes.

## Background

Cigarettes were the most popular tobacco product in the twentieth century, and the most deadly. The risks of cigarette smoking are related to the amount of smoke inhaled and the duration (years, decades) of exposure, and the death toll from cigarette smoking is appalling. For the past 50 years, the American cancer endemic has been heavily influenced by one disease, cancer of the lung, owing to one dominant lifestyle factor—cigarette smoking.

Cigar use also involves exposure to smoke, but much less is known about its health effects. One reason is the low prevalence of cigar smoking and infrequent collection of cigar information in national surveys. For example, the prevalence of cigar use in 2015 among American adult men and women was 4.1% and 0.6%, respectively [1]. In addition, National Health Interview Surveys (NHIS), the primary instrument used by the Centers for Disease Control and Prevention (CDC) to estimate US cigarette smoking rates, collected information about cigar use in only nine of the last 30 years.

The cigar category consists mainly of two types of products: traditional, regular or premium cigars and cigarillos and little filtered cigars. The first type is larger and contains tightly rolled tobacco wrapped in a tobacco leaf. The second category has been described by the National Health Interview Survey questionnaires since 2015: “Cigarillos are medium cigars that sometimes are sold with plastic or wooden tips” and “sold individually or in packs of 5 or fewer. Little filtered cigars look like cigarettes and are usually brown in color. Like cigarettes, little filtered cigars have a spongy filter and are sold in packs of 20.” [2].

These differences are important. It has been known for decades that exclusive users of traditional cigars and pipes tend to puff, not inhale, the smoke [3], thus limiting systemic exposure to toxic constituents compared with cigarette smoking. A study of pipe smokers demonstrated the biomechanics of puffing, which involves closure of the oropharyngeal isthmus by putting the posterior tongue in contact with the soft palate. [4]. In fact, it was suggested that cigarette smokers might be trained to switch from cigarettes to cigars or pipes, while at the same time switching from inhalation to puffing [4].

In contrast, users of cigarillos and small cigars generally inhale the smoke [5]. These cigar types are more commonly consumed by adults under age 40 who are less educated and lower income than regular cigar smokers. They are also more likely to be consumed daily, in larger numbers, and also concurrently with cigarettes [6].

Two follow-up mortality studies of cigar smoking have recently been published. The first was authored by FDA investigators [7], and it involved 1139 current cigar smokers in US Census Bureau surveys in 1985 and 1992–2011, stratified by daily and non-daily use. Multivariable adjusted results showed significantly elevated all-cause mortality for daily users (hazard ratio, HR = 1.22), in addition to increases in smoking-related cancers, lung cancer and chronic obstructive pulmonary disease.

The second study was from investigators at the National Cancer Institute, and it involved 728 exclusive current cigar smokers from selected NHIS years linked to the National Death Index [8]. The authors did not report significant increased mortality for all causes or specific diseases. However, that study included adults of any age, which includes many younger participants (less than age 40 years) who are at little risk of death from any causes for at least 20 years of follow-up.

We conducted a similar follow-up mortality study of cigar-smoking NHIS participants, but we limited our study population to men 40–79 years of age. In addition, we utilized categories of cigarette and cigar smoking that accommodate dual and former use of both products, which partially addresses some of the problems specific to cigars.

## Methods

### Data

This study used pooled files from the Integrated Public Use Microdata Series (IPUMS) for NHIS surveys with information on cigarette and cigar smoking (1987, 1991, 1992, 1998, 2000, 2005 and 2010) and linked to the National Death Index (NDI) [2] to obtain follow-up from year of interview to year of death or end of follow-up, December 31, 2015. Details of the data linkage can be found in our previous study [9].

### Study population

The total number of participants for all NHIS survey years was 752,153. Our analyses were restricted a priori to men age 40–79 years (n = 52,710) since cigars are rarely used by women [6], and at younger ages tobacco use may be less stable and deaths are rare [10].

About 97% of these men (n = 51,062) were eligible for mortality linkage. Men who died the same year as their survey enrollment (n = 366) accrued no person-years, so they were not eligible for analysis. We also excluded men with incomplete information on cigarette or cigar use or demographic characteristics (i.e., race/ethnicity, education and marital status), so the final sample for our analyses was 43,202 men (age 40–59 years, n = 27,229 and age 50–79 years, n = 15,973) with 588,761 person-years and 14,657 deaths.

### Measures

#### Tobacco status

The main predictor of mortality outcomes was cigarette and cigar smoking status at the survey enrollment. We used standard definitions for cigarette smokers. Never cigarette smokers had never smoked 100 cigarettes in their lifetime. Current cigarette smokers had smoked at least 100 cigarettes and smoked every day or some days at the time of the survey. Former cigarette smokers had smoked 100 cigarettes but did not smoke at the time of the survey.

During the time frame of this study, NHIS surveys collected little or no information about cigar types [2]. Prior to 2000, NHIS surveys asked respondents “Have you ever smoked cigars?” The NHIS 2000 survey asked “Have you ever smoked a cigar?” and added an instruction to “Include small, thin, cigars called 'cigarillos,' 'puritos' or 'chicos,' that are wrapped in tobacco leaf rather than paper, and are made by machine or handrolled.” In 2005 and 2010, the NHIS asked “Have you ever smoked a cigar EVEN ONE TIME?” In all years, participants were then asked “Have you smoked at least 50 cigars in your entire life?” [2].

We defined never cigar smokers as those who had never smoked at least 50 cigars in their lifetime. Current cigar smokers had smoked at least 50 cigars and smoked every day or some days at the time of the survey. Former cigar smokers had smoked at least 50 cigars but did not smoke at the time of the survey.

Next, we constructed 9 categories using cigarette and cigar status: 1) never smokers (never cigarette or cigar), 2) never cigarette and current cigar (i.e., exclusive cigar), 3) never cigarette and former cigar, 4) current cigarette and never cigar (i.e., exclusive cigarette), 5) current cigarette and cigar (i.e., dual users), 6) current cigarette and former cigar, 7) former cigarette and never cigar, 8) former cigarette and current cigar, and 9) former for both products.

#### Individual characteristics

We included the following characteristics as confounders: age, race/ethnicity (non-Hispanic white, non-Hispanic black, other), marital status (never married, married, divorced/separated, widowed), educational attainment (< high school, high school, some college, college and higher), family income ($0-$34,999, $35,000-$74,999, ≥ $75,000), region of residence (Northeast, South, Midwest, West) and survey year.

Age, race/ethnicity, socioeconomic and marital status are well established factors in adult mortality studies [11]. For example, Hispanics have lower adult mortality rates than non-Hispanic whites even though they have more disadvantaged conditions [11, 12]. There is substantial evidence that marriage is associated with lower mortality [13–15]. For example, non-married white men have elevated risks for the mortality from all-causes, cardiovascular diseases, cancers and other diseases than married white men, especially at ages 45–64 years [15].

A recent study using the NHIS surveys linked to the National Death Index suggested that education has an inverse effect on mortality, but only after middle age, around 55 years [16]. Similarly, there is evidence in the literature, suggesting that mortality differs by level of income [11, 17]. Specifically, people with lower income have higher mortality than those with higher income, controlling for health status [17]. Mortality rates vary by geographic locations [18, 19], and the inclusion of survey years was an attempt to capture any variations due to unobserved characteristics.

#### Mortality outcomes

We examined all-cause and cause-specific mortality from heart diseases (I00-I09, I11, I13 and I20-I51), malignant neoplasms (C00-C97), chronic lower respiratory diseases (J40-J47) and cerebrovascular disease (I60-I69). In addition, we combined those diseases with diabetes mellitus (E10–E14) and influenza/pneumonia (J09–J18) to make a category called smoking-related diseases similar to, but somewhat broader than those recognized by the Surgeon General [20]. This category was mutually exclusive and exhaustive with respect to all other causes, which consisted of accidents (V01-X59,Y85-Y86), Alzheimer's disease (G30), nephritis, nephrotic syndrome and nephrosis (N00-N07, N17-N19, N25-N27) and all other residual causes [9].

### Statistical analysis

Cox proportional hazards models were used to examine the associations between cigar status and mortality outcomes based on underlying causes of death from the 10th revision of International Statistical Classification of Diseases and Related Health Problems (ICD-10) [21], reported as hazard ratios (HRs, with 95% confidence intervals, CI) with never smokers as the referent group. Follow-up, in years between survey enrollment and death or survival until December 31, 2015, ranged from 1 to 28 years (mean = 13.6 years; median = 14 years, standard deviation = 7.3 years).

We estimated HRs adjusted for age, race/ethnicity, marital status, education, income, region and survey year. We included an indicator for missing family income**,** and we applied sample weights adjusted for NDI linkage eligibility in all regression models. Results are reported separately for younger (ages 40–59 years), older (age 60–79 years) and pooled age groups.

## Results

### Descriptive statistics

Table 1 contains demographic and health characteristics of all men in the study (n = 43,202), according to cigarette and cigar status. There were 2168 current cigar smokers; the largest number (n = 882, 41%) were also current cigarette smokers, followed by former (n = 686, 31%) and never cigarette smokers (i.e., exclusive cigar, n = 600, 28%). Compared with never users, current cigar smokers were more likely to be non-Hispanic white, divorced/separated and obese.

**Table 1:** Demographic characteristics (unweighted percentages) of men age 40–79 years enrolled in NHIS 1987, 1991–92, 1998, 2000, 2005 and 2010, according to cigarette and cigar use

	Never cigarette smokers			Current cigarette smokers			Former cigarette smokers			Total
	Never cigar smokers	Current cigar smokers	Former cigar smokers	Never cigar smokers	Current cigar smokers	Former cigar smokers	Never cigar smokers	Current cigar smokers	Former cigar smokers	
N	14,990	600	957	8345	882	1672	11,162	686	3908	43,202
Age in years	54	54	60	53	52	55	59	56	61	56
Race/ethnicity										
Non-Hispanic White	69	78	86	69	76	85	76	87	89	74
Non-Hispanic Black	13	15	9	18	14	10	10	8	6	12
Hispanic and other race	18	8	6	14	10	6	13	5	5	14
Marital status										
Never married	13	10	7	14	12	8	7	8	5	11
Married	67	62	71	52	51	56	69	67	72	64
Divorced/separated	16	20	15	29	31	29	17	20	16	20
Widowed	4	8	7	5	5	7	7	6	8	6
Educational attainment										
Less than high school	16	18	17	26	27	26	22	13	22	21
High school	27	25	28	37	34	37	31	32	32	31
Some college	21	22	21	23	24	23	22	22	22	22
College and higher	36	36	35	14	15	15	24	33	24	26
Family income										
$0–34,999	42	40	42	55	55	56	47	38	48	47
$35,000–74,999	32	35	36	29	26	29	32	34	34	31
$75,000 +	19	19	14	8	11	7	13	20	11	14
Missing income	8	7	9	8	8	8	9	7	8	8
Region										
Northeast	20	25	21	18	16	15	20	22	19	19
South	34	30	35	38	39	35	35	30	34	35
Midwest	23	27	27	24	23	29	23	31	27	24
West	23	18	18	19	22	22	23	17	20	22

Participants with missing cigar status, race/ethnicity, educational attainment and marital status are excluded

### Smoking status and mortality

**Fig. 1:**
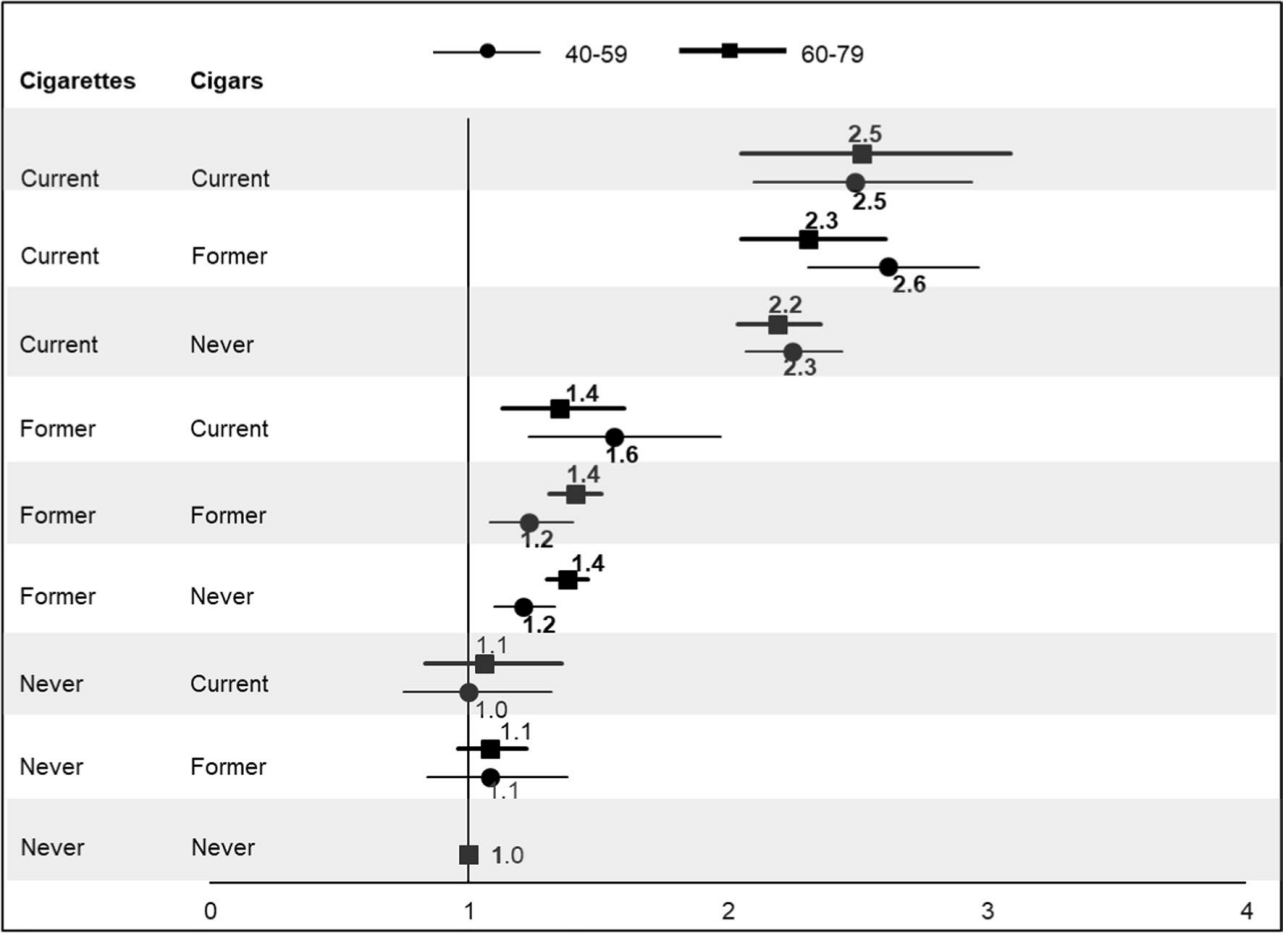
Title: Hazard ratios for all-cause mortality among men age 40–79 years, according to cigarette and cigar use. Numbers are point estimates; bold represents statistically significant at the *p* ≤ 0.05 level. Horizontal lines represent 95% confidence interval

Figure 1 presents the adjusted HRs for mortality from all causes among men who were cigarette and/or cigar smokers compared with never users, according to age group. This was elevated 10% among older current and former exclusive cigar smokers but was not statistically significant. Current cigarette smokers, regardless of cigar status, had the highest HRs, ranging from 2.19 to 2.62, all statistically significant. Former cigarette smokers generally had significant excess all-cause mortality, ranging from 21 to 56%.

**Fig. 2:**
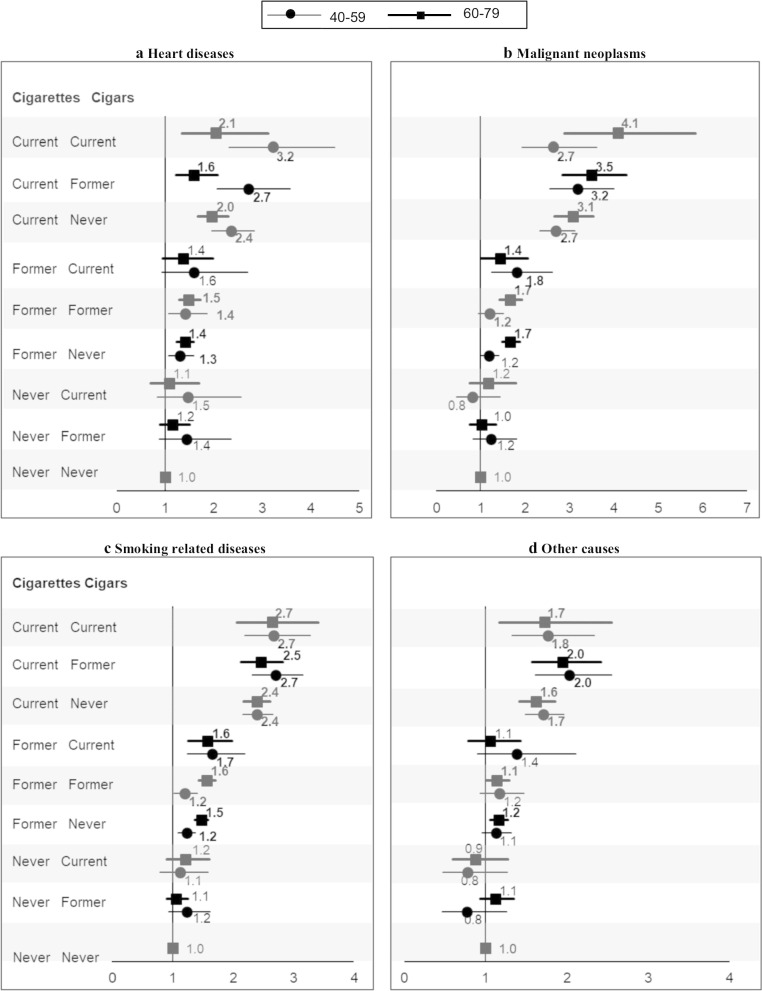
Title: Hazard ratios for mortality from selected diseases among men age 40–79 years, according to cigarette and cigar smoking status. Numbers are point estimates; bold represents statistically significant at the *p* ≤ 0.05 level. Horizontal lines represent 95% confidence interval

Figure 2 shows that current and former exclusive cigar smokers had about a 45% increase in heart disease mortality in the younger age group and a 9% to 16% increase in the older group, none of which were statistically significant (See Additional file 1: Table 1 for all results). For malignant neoplasms, smoking-related diseases and other causes, the results were mixed. For example, younger current exclusive cigar smokers had about 18% lower mortality (HR = 0.82) for malignant neoplasms, but they had 12% increased mortality for smoking-related diseases (HR = 1.12). Again, none of the mixed results were statistically significant. It is noteworthy that current and former cigarette smokers also had significantly higher mortality from other causes, which was not seen in exclusive cigar smokers.

In contrast, current cigarette smokers, regardless of their cigar use and age, had significantly elevated mortality for heart disease (HRs from 1.59 to 3.23), malignant neoplasms (HRs from 2.65 to 4.11), smoking-related diseases (HRs from 2.39 to 2.71), and other causes (HRs from 1.62 to 2.03), compared with never smokers. Similarly, we observed some evidence among former cigarette smokers in both age groups of increased mortality from heart diseases, malignant neoplasms, smoking-related diseases and other causes.

In addition, the HRs from chronic lower respiratory diseases (Additional file 1: Table 1) were very large among current cigarette smokers (HRs from 8 to 45). Even though these HRs are statistically significant, they have very wide confidence intervals due to small numbers of deaths in both the exposed and referent groups. Thus, these estimates are unstable. Similarly, the HRs for cerebrovascular disease (Supplemental Table 1) generated wide confidence intervals. Only younger current exclusive cigarette smokers had statistically significant elevated mortality (HR = 1.99).

Similarly, we observed some evidence among former cigarette smokers in both age groups of increased mortality from heart diseases, malignant neoplasms, chronic lower respiratory diseases, smoking-related diseases and other causes. However, no increase was found for cerebrovascular disease mortality.

## Discussion

The main finding of this study is that exclusive male cigar smokers age 40 + years had no statistically significant increased mortality from all causes, heart diseases, malignant neoplasms, cerebrovascular disease, smoking-related diseases or other causes. In contrast, the mortality experience of dual users of cigars and cigarettes and cigar smokers who formerly used cigarettes is similar to exclusive cigarette smokers.

As noted in the introduction, exclusive smokers of cigars prefer traditional products, sometimes called premium, which are smoked less frequently and are puffed, not inhaled. This might play a role in lower mortality compared with dual users and former cigarette smokers, who are more likely to smoke small cigars frequently and inhale the smoke.

Our findings were similar to those from Inoue-Choi et al. [8]. They used restricted NHIS-Linked Mortality Files and found no elevated mortality among exclusive current and former cigar smokers. Current daily cigar smokers had elevated mortality from cancer (HR = 2.40, CI = 1.19 to 4.81). However, the increase was not attributable to oral cavity, esophagus, stomach, colorectal or pancreas cancers. Furthermore, nonsignificant increases in lung and bladder cancer were based on fewer than five deaths.

Another recent study by Christensen et al. [7] used data from the Tobacco Use Supplement to the Current Population Survey (TUS-CPS) linked to the National Longitudinal Mortality Study. It found that exclusive current cigar smokers ages 35–80 years had significantly elevated mortality for all causes (HR = 1.20, CI = 1.03–1.38), all tobacco-related cancers (HR = 1.61, CI = 1.11–2.32) and lung cancer (HR = 3.26, CI = 1.86–5.71). The authors observed that, although most cigar users did not smoke every day, the mortality increases were mainly due to daily users. No excess deaths were found among exclusive former cigar smokers.

Christensen et al. [7] and Inoue-Choi et al. [8] included both men and women, and the latter included all ages. More importantly, following several unanswered inquiries from us (personal communications), the Inoue-Choi et al. reported coding errors requiring republication of their article as a corrigendum [22]. Both studies adjusted for only sociodemographic variables and survey years, whereas our models adjusted for more comprehensive confounders. More importantly, those studies excluded use of other tobacco products, which eliminates 60% of all current cigar smokers from follow-up [23]. Our approach includes the mortality experience of dual current cigar-cigarette users and current-former users of these two products. Our HRs for current and former cigarette smokers, regardless of their cigar use, were similar to those from previous studies by us [9] and others [7, 24, 25]. Dual users had significantly elevated mortality risks for all outcomes except cerebrovascular disease. Former cigarette smokers who smoked cigars at the time of the survey had excess mortality for most causes, but the magnitudes were smaller than current cigarette smokers.

Other factors may be related to the low impact on mortality of cigar smoking in this study. As discussed earlier, male cigar smokers over age 40 who never smoked cigarettes are more likely to be consumers of traditional cigars, which tend to be smoked less frequently and in smaller numbers than cigarillos or filtered cigars [6]. Additionally, differences in risk between cigar and cigarette smokers has been attributed for years to differences in inhalation practices [6, 26, 27]. Although these factors might contribute to exclusive cigar smokers’ lower mortality compared to cigarette smokers, they do not seem to benefit former cigarette smokers who are current cigar smokers, who had elevated mortality for most diseases.

Our results also mirror those cited in a systematic review on cigars and health outcomes by Chang et al. [27], which defined primary cigar smokers as having had no history of cigarette use. The results were organized according to the number of cigars smoked daily and cited two studies reporting all-cause mortality [28, 29]. The first involved 15,000 primary cigar smokers in the American Cancer Society First Cancer Prevention Survey [28]. Primary cigar smokers consuming 1–2 cigars daily had no increased mortality, but those smoking 3–4 and 5 or more had 8% and 17% elevated mortality from all causes, respectively. In the second study of 250,000 government-insured participants, most of whom were World War I veterans, fewer than 5 cigars per day was associated with no significant increase [29].

Similar results for other diseases related to primary smokers of 1–2 cigars per day were cited in the review by Chang et al. [27]. For stomach, pancreas and bladder cancer, elevated risks were based on very small numbers of deaths and not statistically significant. Some cancer estimates were elevated, especially mouth/throat, esophagus, larynx and lung, but none were statistically significant. However, an older study by Shapiro et al. [30] using Cancer Prevention Study II found that cigar smoking men age 30 + years had elevated mortality risk for lung, oral cavity/pharynx, larynx and esophagus cancer.

Chang et al. [27] observed no increased mortality from coronary heart disease, stroke or emphysema among primary smokers of 1–2 cigars per day, but they did note an 80% excess of deaths from aortic aneurysm in the American Cancer Society First Cancer Prevention Survey [30].

The most important limitation of this study is the lack of information about the type(s) of cigar smoked, number, frequency and duration of consumption among current smokers and the number of years since quitting among former smokers. As discussed earlier, we tried to partially account for these deficiencies by distinguishing between exclusive cigar smokers and those with a cigarette smoking history, and by restricting our study to men age 40 + years. In addition, NHIS collects information on the use of cigarettes and cigars only once at survey enrollment for each participant, and information on alcohol use and preexisting chronic conditions (e.g., diabetes, high blood pressure, high cholesterol), which are risk factors for premature death, was not available consistently after 1997 in NHIS. Similarly, there were no consistent data on physical activity and diet. Finally, we have limited statistical power and high standard errors for some outcomes due to the low prevalence of cigar use and small numbers of deaths.

## Conclusions

This study provides limited evidence that male cigar smokers had elevated mortality risks. After accounting for cigarette smoking, we found a paucity of effects consistent with other studies, which may be related to patterns and frequency among exclusive primary cigar smokers.

## Supplementary information

**Additional file 1**. Hazard ratios of all-cause mortality associated with cigarette and cigar use status among men 40–79 years in NHIS 1987, 1991, 1992, 1998, 2000, 2005, and 2010.

## Data Availability

The datasets analyzed during the current study are available in the IPUMS repository at http://doi.org/10.18128/D070.V6.3

## Abbreviations

NHIS: National health interview survey
HR: Hazard ratio
CI: Confidence interval
IPUMS: Integrated public use microdata series
NDI: National Death Index
ICD: International classification of diseases and related health problems
BMI: Body mass index
CPS: Cancer prevention study
TUS-CPS: Tobacco use supplement to the current population survey
