# Supplementary material for: Mortality among male cigar and cigarette smokers in the USA
Source: Harm Reduct J. 2021 Jan 7;18:7. doi: 10.1186/s12954-020-00446-4 (PMC7789747; doi:10.1186/s12954-020-00446-4)
Supplement: Supplementary file 1 — Additional file 1. Hazard ratios of all-cause mortality associated with cigarette and cigar use status among men 40–79 years in NHIS 1987, 1991, 1992, 1998, 2000, 2005, and 2010. [file 12954_2020_446_MOESM1_ESM.docx]

**Supplemental Table 1**. Hazard ratios of all-cause mortality associated with cigarette and cigar use status among men 40-79 years in NHIS 1987, 1991, 1992, 1998, 2000, 2005, and 2010

|  |  | Age 40-59 | | Age 60-79 | | Age 40-79 | |
| --- | --- | --- | --- | --- | --- | --- | --- |
|  |  | # of death | HR | # of death | HR | # of death | HR |
| **1. All causes** |  |  |  |  |  |  |  |
| **Cigarette** | **Cigar** |  |  |  |  |  |  |
|  |  |  |  |  |  |  |  |
| Current | Current | 218 | **2.49** | 129 | **2.52** | 347 | **2.53** |
|  |  |  | **[2.10,2.94]** |  | **[2.05,3.09]** |  | **[2.22,2.88]** |
|  |  |  |  |  |  |  |  |
| Current | Former | 405 | **2.62** | 416 | **2.31** | 821 | **2.45** |
|  |  |  | **[2.31,2.97]** |  | **[2.05,2.61]** |  | **[2.24,2.67]** |
|  |  |  |  |  |  |  |  |
| Current | Never | 1874 | **2.25** | 1402 | **2.19** | 3276 | **2.25** |
|  |  |  | **[2.07,2.44]** |  | **[2.04,2.36]** |  | **[2.13,2.38]** |
|  |  |  |  |  |  |  |  |
| Former | Current | 89 | **1.56** | 149 | **1.35** | 238 | **1.40** |
|  |  |  | **[1.23,1.97]** |  | **[1.13,1.60]** |  | **[1.21,1.61]** |
|  |  |  |  |  |  |  |  |
| Former | Former | 349 | **1.23** | 1402 | **1.41** | 1751 | **1.37** |
|  |  |  | **[1.08,1.40]** |  | **[1.31,1.51]** |  | **[1.29,1.45]** |
|  |  |  |  |  |  |  |  |
| Former | Never | 991 | **1.21** | 3243 | **1.38** | 4234 | **1.33** |
|  |  |  | **[1.10,1.33]** |  | **[1.30,1.46]** |  | **[1.27,1.39]** |
|  |  |  |  |  |  |  |  |
| Never | Current | 56 | 1.00 | 120 | 1.06 | 176 | 1.02 |
|  |  |  | [0.75,1.32] |  | [0.83,1.36] |  | [0.86,1.23] |
|  |  |  |  |  |  |  |  |
| Never | Former | 78 | 1.08 | 310 | 1.08 | 388 | 1.08 |
|  |  |  | [0.84,1.38] |  | [0.96,1.22] |  | [0.97,1.21] |
|  |  |  |  |  |  |  |  |
| Never | Never | 1227 | Ref. | 2199 | Ref. | 3426 | Ref. |
|  |  |  |  |  |  |  |  |
| **2. Heart Diseases** |  |  |  |  |  |  |  |
| **Cigarette** | **Cigar** |  |  |  |  |  |  |
|  |  |  |  |  |  |  |  |
| Current | Current | 53 | **3.23** | 26 | **2.05** | 79 | **2.70** |
|  |  |  | **[2.32,4.50]** |  | **[1.35,3.13]** |  | **[2.09,3.49]** |
|  |  |  |  |  |  |  |  |
| Current | Former | 85 | **2.72** | 77 | **1.59** | 162 | **2.04** |
|  |  |  | **[2.07,3.58]** |  | **[1.22,2.09]** |  | **[1.68,2.46]** |
|  |  |  |  |  |  |  |  |
| Current | Never | 367 | **2.36** | 302 | **1.97** | 669 | **2.14** |
|  |  |  | **[1.96,2.84]** |  | **[1.68,2.31]** |  | **[1.91,2.41]** |
|  |  |  |  |  |  |  |  |
|  |  |  |  |  |  |  |  |
| Former | Current | 17 | 1.60 | 37 | 1.37 | 54 | 1.42 |
|  |  |  | [0.94,2.71] |  | [0.95,1.99] |  | [1.05,1.93] |
|  |  |  |  |  |  |  |  |
| Former | Former | 78 | **1.41** | 345 | **1.49** | 423 | **1.49** |
|  |  |  | **[1.07,1.87]** |  | **[1.29,1.73]** |  | **[1.31,1.69]** |
|  |  |  |  |  |  |  |  |
| Former | Never | 210 | **1.31** | 759 | **1.41** | 969 | **1.38** |
|  |  |  | **[1.07,1.60]** |  | **[1.24,1.59]** |  | **[1.24,1.53]** |
|  |  |  |  |  |  |  |  |
| Never | Current | 15 | 1.47 | 30 | 1.09 | 45 | 1.21 |
|  |  |  | [0.84,2.56] |  | [0.70,1.70] |  | [0.85,1.71] |
|  |  |  |  |  |  |  |  |
| Never | Former | 20 | 1.44 | 76 | 1.16 | 96 | 1.23 |
|  |  |  | [0.88,2.36] |  | [0.90,1.51] |  | [0.98,1.55] |
|  |  |  |  |  |  |  |  |
| Never | Never | 231 | Ref. | 515 | Ref. | 746 | Ref. |
|  |  |  |  |  |  |  |  |
| **3. Malignant neoplasms** | |  |  |  |  |  |  |
| **Cigarette** | **Cigar** |  |  |  |  |  |  |
|  |  |  |  |  |  |  |  |
| Current | Current | 62 | **2.65** | 52 | **4.11** | 114 | **3.31** |
|  |  |  | **[1.93,3.62]** |  | **[2.89,5.84]** |  | **[2.63,4.18]** |
|  |  |  |  |  |  |  |  |
| Current | Former | 144 | **3.20** | 144 | **3.50** | 288 | **3.42** |
|  |  |  | **[2.56,4.00]** |  | **[2.85,4.29]** |  | **[2.94,3.98]** |
|  |  |  |  |  |  |  |  |
| Current | Never | 651 | **2.70** | 438 | **3.08** | 1089 | **2.98** |
|  |  |  | **[2.34,3.13]** |  | **[2.66,3.55]** |  | **[2.69,3.30]** |
|  |  |  |  |  |  |  |  |
| Former | Current | 35 | **1.81** | 36 | **1.44** | 71 | **1.59** |
|  |  |  | **[1.25,2.62]** |  | **[1.01,2.07]** |  | **[1.22,2.06]** |
|  |  |  |  |  |  |  |  |
| Former | Former | 110 | 1.20 | 351 | **1.66** | 461 | **1.51** |
|  |  |  | [0.95,1.52] |  | **[1.43,1.93]** |  | **[1.34,1.71]** |
|  |  |  |  |  |  |  |  |
| Former | Never | 301 | 1.19 | 841 | **1.67** | 1142 | **1.51** |
|  |  |  | [1.01,1.41] |  | **[1.48,1.89]** |  | **[1.37,1.66]** |
|  |  |  |  |  |  |  |  |
| Never | Current | 14 | 0.82 | 27 | 1.17 | 41 | 1.00 |
|  |  |  | [0.46,1.44] |  | [0.76,1.80] |  | [0.71,1.41] |
|  |  |  |  |  |  |  |  |
| Never | Former | 31 | 1.23 | 60 | 1.02 | 91 | 1.05 |
|  |  |  | [0.83,1.81] |  | [0.76,1.36] |  | [0.83,1.33] |
| Never | Never | 365 | Ref. | 460 | Ref. | 825 | Ref. |
|  |  |  |  |  |  |  |  |
| **4. Smoking related diseases** | |  |  |  |  |  |  |
| **Cigarette** | **Cigar** |  |  |  |  |  |  |
|  |  |  |  |  |  |  |  |
| Current | Current | 152 | **2.68** | 95 | **2.65** | 247 | **2.73** |
|  |  |  | **[2.19,3.28]** |  | **[2.06,3.41]** |  | **[2.33,3.19]** |
|  |  |  |  |  |  |  |  |
| Current | Former | 287 | **2.71** | 295 | **2.46** | 582 | **2.60** |
|  |  |  | **[2.32,3.16]** |  | **[2.13,2.83]** |  | **[2.34,2.89]** |
|  |  |  |  |  |  |  |  |
| Current | Never | 1284 | **2.39** | 1011 | **2.39** | 2295 | **2.44** |
|  |  |  | **[2.16,2.66]** |  | **[2.17,2.62]** |  | **[2.28,2.62]** |
|  |  |  |  |  |  |  |  |
| Former | Current | 61 | **1.65** | 103 | **1.58** | 164 | **1.59** |
|  |  |  | **[1.24,2.19]** |  | **[1.25,1.98]** |  | **[1.33,1.91]** |
|  |  |  |  |  |  |  |  |
| Former | Former | 232 | **1.20** | 952 | **1.56** | 1184 | **1.48** |
|  |  |  | **[1.02,1.41]** |  | **[1.43,1.71]** |  | **[1.37,1.60]** |
|  |  |  |  |  |  |  |  |
| Former | Never | 647 | **1.23** | 2152 | **1.48** | 2799 | **1.41** |
|  |  |  | **[1.09,1.38]** |  | **[1.37,1.59]** |  | **[1.33,1.50]** |
|  |  |  |  |  |  |  |  |
| Never | Current | 38 | 1.12 | 75 | 1.21 | 113 | 1.16 |
|  |  |  | [0.79,1.59] |  | [0.90,1.61] |  | [0.92,1.47] |
|  |  |  |  |  |  |  |  |
| Never | Former | 59 | 1.23 | 175 | 1.06 | 234 | 1.09 |
|  |  |  | [0.93,1.62] |  | [0.90,1.26] |  | [0.94,1.27] |
|  |  |  |  |  |  |  |  |
| Never | Never | 726 | Ref. | 1277 | Ref. | 2003 | Ref. |
|  |  |  |  |  |  |  |  |
| **5. Other causes** |  |  |  |  |  |  |  |
| **Cigarette** | **Cigar** |  |  |  |  |  |  |
|  |  |  |  |  |  |  |  |
| Current | Current | 64 | **1.77** | 33 | **1.73** | 97 | **1.76** |
|  |  |  | **[1.33,2.34]** |  | **[1.17,2.55]** |  | **[1.40,2.21]** |
|  |  |  |  |  |  |  |  |
| Current | Former | 118 | **2.03** | 121 | **1.95** | 239 | **1.94** |
|  |  |  | **[1.61,2.55]** |  | **[1.57,2.42]** |  | **[1.66,2.27]** |
|  |  |  |  |  |  |  |  |
| Current | Never | 580 | **1.71** | 386 | **1.62** | 966 | **1.67** |
|  |  |  | **[1.49,1.96]** |  | **[1.42,1.86]** |  | **[1.53,1.84]** |
|  |  |  |  |  |  |  |  |
|  |  |  |  |  |  |  |  |
| Former | Current | 28 | 1.38 | 46 | 1.06 | 74 | 1.14 |
|  |  |  | [0.90,2.11] |  | [0.79,1.43] |  | [0.89,1.46] |
|  |  |  |  |  |  |  |  |
| Former | Former | 116 | 1.17 | 449 | **1.14** | 565 | **1.14** |
|  |  |  | [0.93,1.47] |  | **[1.01,1.29]** |  | **[1.02,1.26]** |
|  |  |  |  |  |  |  |  |
| Former | Never | 343 | 1.13 | 1083 | **1.16** | 1426 | **1.14** |
|  |  |  | [0.96,1.32] |  | **[1.06,1.28]** |  | **[1.05,1.23]** |
|  |  |  |  |  |  |  |  |
| Never | Current | 18 | 0.78 | 45 | 0.88 | 63 | 0.84 |
|  |  |  | [0.48,1.27] |  | [0.60,1.28] |  | [0.63,1.11] |
|  |  |  |  |  |  |  |  |
| Never | Former | 19 | 0.77 | 135 | 1.12 | 154 | 1.08 |
|  |  |  | [0.47,1.26] |  | [0.93,1.35] |  | [0.91,1.28] |
|  |  |  |  |  |  |  |  |
| Never | Never | 496 | Ref. | 914 | Ref. | 1410 | Ref. |
|  |  |  |  |  |  |  |  |
| **6. Chronic lower respiratory diseases** | |  |  |  |  |  |  |
| **Cigarette** | **Cigar** |  |  |  |  |  |  |
|  |  |  |  |  |  |  |  |
| Current | Current | 23 | **44.67** | 10 | **7.98** | 33 | **16.50** |
|  |  |  | **[19.01,104.98]** |  | **[4.01,15.88]** |  | **[10.64,25.58]** |
|  |  |  |  |  |  |  |  |
| Current | Former | 28 | **24.87** | 42 | **9.29** | 70 | **11.95** |
|  |  |  | **[10.76,57.45]** |  | **[6.11,14.13]** |  | **[8.36,17.08]** |
|  |  |  |  |  |  |  |  |
| Current | Never | 114 | **25.31** | 155 | **10.00** | 269 | **12.48** |
|  |  |  | **[11.74,54.58]** |  | **[7.20,13.88]** |  | **[9.30,16.74]** |
|  |  |  |  |  |  |  |  |
| Former | Current | 1 | 2.86 | 10 | **4.14** | 11 | **3.97** |
|  |  |  | [0.35,23.34] |  | **[2.09,8.19]** |  | **[2.08,7.59]** |
|  |  |  |  |  |  |  |  |
| Former | Former | 13 | **5.50** | 80 | **3.27** | 93 | **3.68** |
|  |  |  | **[2.18,13.85]** |  | **[2.29,4.68]** |  | **[2.64,5.14]** |
|  |  |  |  |  |  |  |  |
| Former | Never | 36 | **6.64** | 217 | **3.76** | 253 | **4.27** |
|  |  |  | **[2.95,14.97]** |  | **[2.75,5.14]** |  | **[3.19,5.71]** |
|  |  |  |  |  |  |  |  |
| Never | Current | 1 | 2.88 | 5 | **2.53** | 6 | **2.50** |
|  |  |  | [0.35,23.51] |  | **[1.01,6.38]** |  | **[1.07,5.81]** |
|  |  |  |  |  |  |  |  |
| Never | Former | 0 | n/a | 5 | 0.80 | 5 | 0.77 |
|  |  |  |  |  | [0.32,2.01] |  | [0.31,1.93] |
|  |  |  |  |  |  |  |  |
| Never | Never | 7 | Ref. | 49 | **Ref.** | 56 | **Ref.** |
| **7. Cerebrovascular disease** | |  |  |  |  |  |  |
| **Cigarette** | **Cigar** |  |  |  |  |  |  |
|  |  |  |  |  |  |  |  |
| Current | Current | 5 | 1.07 | 4 | 1.01 | 9 | 1.00 |
|  |  |  | [0.39,2.98] |  | [0.37,2.81] |  | [0.49,2.04] |
|  |  |  |  |  |  |  |  |
| Current | Former | 10 | 1.46 | 13 | 1.12 | 23 | 1.22 |
|  |  |  | [0.70,3.06] |  | [0.60,2.09] |  | [0.76,1.96] |
|  |  |  |  |  |  |  |  |
| Current | Never | 67 | **1.99** | 62 | 1.35 | 129 | **1.59** |
|  |  |  | **[1.28,3.10]** |  | [0.96,1.89] |  | **[1.23,2.05]** |
|  |  |  |  |  |  |  |  |
| Former | Current | 2 | 0.81 | 10 | 1.58 | 12 | 1.43 |
|  |  |  | [0.19,3.42] |  | [0.80,3.14] |  | [0.77,2.66] |
|  |  |  |  |  |  |  |  |
| Former | Former | 17 | 1.55 | 79 | 1.24 | 96 | 1.32 |
|  |  |  | [0.84,2.85] |  | [0.92,1.69] |  | [1.00,1.73] |
|  |  |  |  |  |  |  |  |
| Former | Never | 41 | 1.36 | 158 | 1.03 | 199 | 1.10 |
|  |  |  | [0.84,2.19] |  | [0.80,1.33] |  | [0.88,1.37] |
|  |  |  |  |  |  |  |  |
| Never | Current | 3 | 2.30 | 6 | 1.06 | 9 | 1.26 |
|  |  |  | [0.59,8.93] |  | [0.43,2.59] |  | [0.60,2.67] |
|  |  |  |  |  |  |  |  |
| Never | Former | 4 | 1.31 | 16 | 0.65 | 20 | 0.76 |
|  |  |  | [0.45,3.81] |  | [0.38,1.13] |  | [0.46,1.24] |
|  |  |  |  |  |  |  |  |
| Never | Never | 43 | Ref. | 133 | Ref. | 176 | Ref. |
|  |  |  |  |  |  |  |  |
| N |  |  | 27229 |  | 15973 |  | 43202 |

[95% confidence interval]

**Bold numbers** denote statistically significant at the p < 0.05 level.

All results are weighted and adjusted for age, race/ethnicity, marital status, education, income, region, and survey years. Participants with missing tobacco status, race/ethnicity, educational attainment, and marital status are excluded.

Smoking related diseases: diseases of heart, malignant neoplasms, chronic lower respiratory diseases, cerebrovascular diseases, diabetes mellitus, and influenza and pneumonia.

Other causes: accidents, Alzheimer's disease, Nephritis, nephrotic syndrome and nephrosis and all other causes.
